# Diversity of endophytic fungal community in *Huperzia serrata* from different ecological areas and their correlation with Hup A content

**DOI:** 10.1186/s12866-022-02605-y

**Published:** 2022-08-05

**Authors:** Bo Pang, Dengpan Yin, Yufeng Zhai, Anguo He, Linlin Qiu, Qiao Liu, Nan Ma, Hongjun Shen, Qiaojun Jia, Zongsuo Liang, Dekai Wang

**Affiliations:** 1 College of Life Sciences and Medicine, Zhejiang Sci-Tech University, Hangzhou, 310018 Zhejiang China; 2 Administration of Zhejiang Dapanshan National Nature Reserve, Pan’an, Zhejiang, 322300 China; 3 Ningbo Delai Medicinal Material Planting Co, Zhejiang 315444 Ltd Ningbo, China

**Keywords:** *Huperzia serrata*, Endophytic fungi, Hup A, Community diversity, High throughput sequencing

## Abstract

**Background:**

Huperzine A (Hup A) has attracted considerable attention as an effective therapeutic candidate drug used to treat Alzheimer’s disease. Whereas, the production of Hup A from wild plants faced a major challenge, which is the wild *Huperzia Serrata* harbor a low Hup A content, has a long-life cycle, and has a small yield. At present, several reports showed that Hup A is produced by various endophytic fungal strains isolated from *H. serrata*, thereby providing an alternative method to produce the compound and reduce the consumption of this rare and endangered plant. However, till now, very few comprehensive studies are available on the biological diversity and structural composition of endophytic fungi and the effects of endophytic fungi on the Hup A accumulation in *H. serrata*.

**Results:**

In this research, the composition and diversity of fungal communities in *H. serrata* were deciphered based on high-throughput sequencing technology of fungal internal transcribed spacer regions2 (ITS2). The correlation between endophytic fungal community and Hup A content was also investigated. Results revealed that the richness and the diversity of endophytic fungi in *H. serrata* was various according to different tissues and different ecological areas. The endophytic fungal communities of *H. serrata* exhibit species-specific, ecological-specific, and tissue-specific characteristics. There are 6 genera (Ascomycota_unclassified, *Cyphellophora,* Fungi_unclassified, *Sporobolomyces,* and Trichomeriaceae_unclassified) were significantly positively correlated with Hup A content in all two areas, whereas, there are 6 genera (*Auricularia, Cladophialophora, Cryptococcus, Mortierella,* and *Mycena*) were significantly negatively correlated with Hup A content of in all two areas.

**Conclusions:**

This study indicated a different composition and diverse endophytic fungal communities in *H. serrata* from different organs and ecological areas. The current study will provide the realistic basis and theoretical significance for understanding the biological diversity and structural composition of endophytic fungal communities in *H. serrata*, as well as providing novel insights into the interaction between endophytic fungi and Hup A content.

## Background

Huperzine A (Hup A) is a highly active alkaloid extracted and isolated from the herbal plant *Huperzia serrata*, and has been successfully used to treat myasthenia gravis and Alzheimer’s disease (AD) as a potent, reversible, and selective acetylcholinesterase (AChE) inhibitor [1–5]. Unfortunately, the Hup A content in wild *H. serrata* is relatively low, and the content of the compound in the dried herb is less than 0.025% [6, 7]. Furthermore, *H. serrata* grows very slowly and has a long-life cycle, and a small yield can be obtained, as well as hardly be cultivated under natural conditions currently [4]. Thus, the current yield of wild *H. serrata* is far from meeting the increasing demand for Hup A in clinical treatment. Which also led to the scarcity of raw materials and overexploitation of wild resources. As a result, *H*. *serrata* is currently listed as a Level II nationally protected plant species in China [8].

Endophytic fungi reside within the tissues of almost all living plants, without causing any apparent harm or pathogenic infection to their host, and play critical roles in plant growth, development, fitness improving stress resistance, protecting plants from phytopathogens, and promoting nutrient absorption [9–12]. Furthermore, some endophytic fungi established interaction with their host plants and co-evolved with them. They can produce valuable secondary metabolites like as their hosts, regarded as a novel and important source of bioactive compounds with potential biotechnological applications in the context of agriculture, food, cosmetics, and medicine [13–15]. In recent years, numerous studies have reported that endophytic fungi producing Huperzine A have been isolated from from different *Huperiaceae*, which offers an alternative method to reduce the immense plant harvest-related logistics needed to meet the increasing demand for the Hup A [16–18].

High-throughput sequencing (HTS) technology has the advantages of culture-free, a large amount of information, providing fast and accurate microbial diversity analysis method for microbes, including bacteria, fungi, and arbuscular mycorrhizal fungi (AMF) in various ecosystems [19–22]. Previous studies have reported that diversity and composition of endophytic fungi in cultivated *H. serrata*, and its relevance to the production of Hup A [23, 24]. However, few kinds of research have been conducted on the analysis of the diversity and composition of endophytic fungi when *H. serrata* from different ecological areas and their correlation with Hup A content.

In this study, a high-throughput sequencing method was employed to sequence the ITS region of fungal ribose RNA (rRNA) genes to investigate the fungal community diversity, and composition in root, stem, and leaf samples in *H. serrata* from two different ecological areas. The correlation between Hup A and the endophytic fungal community was also being investigated. Our results will provide new insights for further research on the fungal community of *H. serrata* and their interactions with Hup A production. The current research could help elucidate the ecological function of endophytic fungi and lay a foundation for further research on *H. serrata*.

## Results

### Analysis of the sequencing data

A total of 1,434,573 effective tags of fungal samples were obtained after filtering low-quality and other unsuitable sequences. The number of effective tags per sample ranged from 72,677 to 84,744, and the average number of clean reads was 79,699 per sampled group. The sequence lengths of all samples are mainly concentrated in 200-300 bp and 300-400 bp, accounting for 89.9% and 10.0%, respectively. The quality of the sequencing data was evaluated primarily through the statistics of sequence number, sequence length, GC content, Q20 and Q30 quality values, effective ratio, and other parameters in each sample (Table 1).

**Table 1:** The statistics and quality evaluation of the sequencing data

Sample	Raw_Tags	Valid Tags	Q20(%)	Q30(%)	GC(%)	Valid(%)
CDR_1	87,444	84,744	99.31	97.35	52.54	96.91
CDR_2	85,883	81,799	99.10	96.50	53.74	95.24
CDR_3	80,058	75,929	99.31	97.21	53.11	94.84
CDS_1	85,964	83,838	99.44	97.65	52.09	97.53
CDS_2	82,868	79,893	99.51	97.88	54.59	96.41
CDS_3	82,394	80,274	99.47	97.80	55.60	97.43
CDY_1	81,011	79,162	99.52	97.90	54.67	97.72
CDY_2	83,619	81,254	99.71	98.58	55.33	97.17
CDY_3	85,349	83,548	99.49	97.75	53.94	97.89
QXR_1	80,931	75,692	99.17	96.78	55.47	93.53
QXR_2	82,683	77,556	99.67	98.31	54.46	93.80
QXR_3	81,931	75,342	99.48	97.72	52.79	91.96
QXS_1	85,104	82,224	99.59	98.09	55.67	96.62
QXS_2	83,886	72,677	99.60	98.25	53.26	86.64
QXS_3	81,085	78,158	98.82	95.87	56.20	96.39
QXY_1	84,574	82,311	99.54	97.98	56.57	97.32
QXY_2	84,803	81,718	99.57	98.06	55.37	96.36
QXY_3	80,351	78,454	99.11	96.66	54.11	97.64

**Fig. 1:**
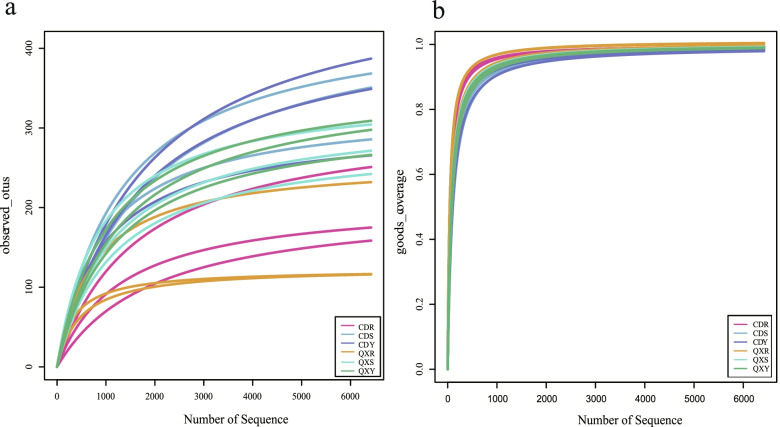
Rarefaction curves of endophytic fungi based on the ITS2 sequences from each group. **a**. Observed OTUs numbers. **b**. Good coverage. Each group was comprised of 3 biological replicates (*n* = 3). CDR, CDS and CDY represent root sample, stem sample and leaf sample from Chengdu respectively; QXR, QXS and QXY represent root sample, stem sample and leaf sample from Qixianhu respectively

The rarefaction curves, displaying the relationship between the number of reads and operational taxonomic units (OTUs) in each sample, exhibited a stable plateau with the increase of the sample size (Fig. 1a). The results indicated that the sequencing depth and the number of OTUs were sufficient for each sample to represent the fungal communities and continue with further analyses. The rarefaction curves also showed that the abundance of fungal community in highest in leaf samples and lowest in root samples. The Good’s coverage values for the eighteen samples ranged from 98.2% to 100% (Fig. 1b), also indicating that the sequencing data confidently reflected the structure of the endophytic fungi community of the samples.

### Taxonomic Analysis of Endophytic Fungi

**Fig. 2:**
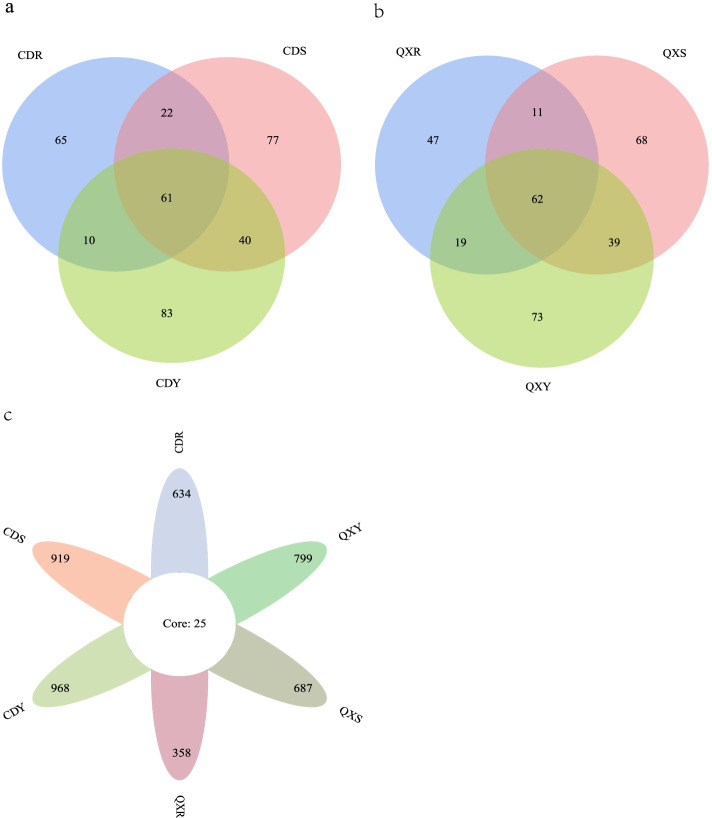
Distribution difference of endophytic fungi in six group from two different ecological areas. Description: The Venn diagram (**a** and **b**) and petal diagram (**c**) based on operational taxonomic units (OTU), which represent common or unique OTUs to a given group

A total of 2,521 operational taxonomic units (OTUs) were obtained from six groups, and 1,829 and 1,288 OTUs were detected in Chengdu samples and Qixianhu samples, respectively. Moreover, 140 OTUs and 102 OTUs were common to Chengdu samples and Qixianhu samples, respectively (Fig. 2a, b). As depicted in the petal diagram, 25 common OTUs were present in each sample’s, indicating that there may be great differences in endophytic fungi between the two areas due to different ecological conditions (Fig. 2c).

**Fig. 3:**
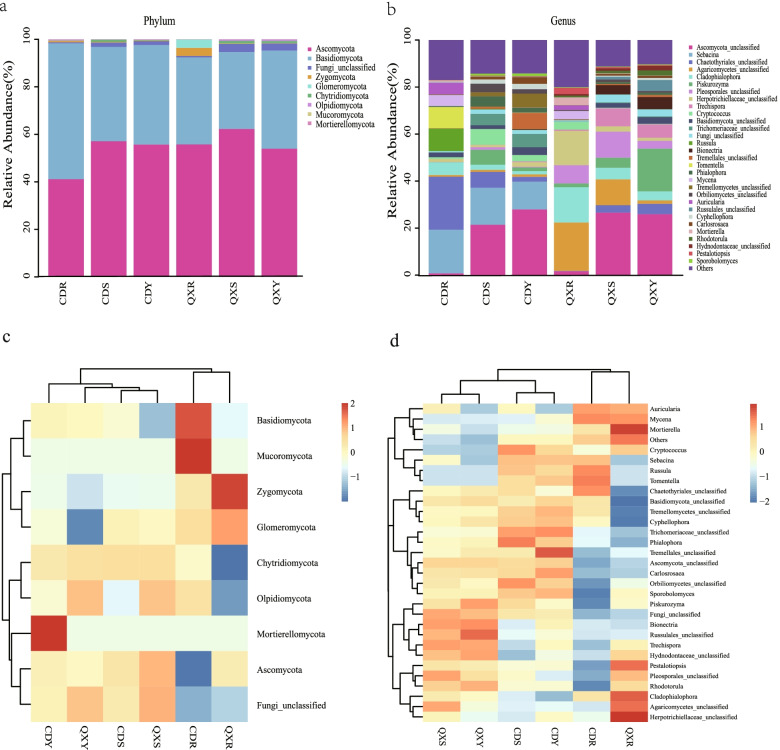
The relative abundance and heatmap of endophytic fungi in the six groups at different taxonomy levels. (**a**): Relative abundance of fungi at the phylum level. (**b**): Relative abundance of fungi at the genus level with a relative abundance of more than 1%. (**c**) and (**d**) The heatmap of shows the absolute abundance of taxa for endophytic fungi at the phylum and genus level, respectively

The taxonomic distribution of endophytic fungi in the roots, stems, and leaves of *H. serrata* is displayed in Fig. 3. After screening out rare OTUs, the remaining OTUs represented 9 phyla, 40 classes, 102 orders, 228 families, and 430 genera, respectively. At the phyla level, the OTUs were assigned to 8 knowns fungi, which were *Ascomycota*, *Basidiomycota*, *Zygomycota*, *Glomeromycota*, *Chytridiomycota*, *Olpidiomycota*, *Mucoromycota*, and *Mortierellomycota*. According to the results of multiple sequence alignment of features sequences of these phyla, the evolutionary tree of feature sequences is constructed (Fig. 3a). Among them, the predominant phylum was *Ascomycota* (54.34%, 41.14%-62.30%), followed by *Basidiomycota* (41.51%, 32.42%-57.17%), Fungi_unclassified (1.83%, 0.49%-3.34%), *Zygomycota* (0.58%, 0.15%-3.41%) and *Glomeromycota* (0.62%, 0%-3.52%). Among them, *Basidiomycota*, *Ascomycota*, *Zygomycota,* and *Olpidiomycota* were found in all tested samples. Otherwise, all *Glomeromycota* were found in root samples and stem samples, but not found in leaf samples from two sites. *Chytridiomycota* and *Mucoromycota* were only found in roots samples from Chengdu (CD), but not found from Qixianhu (QX). In addition, *Mucoromycota* and *Mortierellomycota* were only found in root samples and leaf samples from Chengdu (CD), respectively. At the genus level, a total of 430 distinct fungal genera were identified, and the compositions and proportions of the genera were significantly different among different tissues and different ecological areas. The genus *Ascomycota* was the most abundant in leaf and stem samples (CDY, CDS, QXY, and QXS), with relative abundances ranging from 21.45% to 28%. Whereas, *Ascomycota* genus was relative low abundance in root samples, with the relatively abundance was 0.77% and 1.67% in CDR and QXR, respectively. And *Piskurozyma* genus showed similar features. In contrast, *Cladophialophora* and *Mycena* genera were more abundant in root samples than in leaf and stem samples. It is remarkable to mention that *Sebacina* was the second dominant genus in Chengdu samples (CDR, CDS, and CDY), which account for 18.54%, 15.74%, and 11.76%, respectively, but it was hardly detected in Qixianhu samples (QXR, QXS, and QXY) (Fig. 3b).

The top 9 classes of endophytic fungi in *H. serrata* were selected to make a clustering heatmap, which further indicating that species distributions differed greatly across the three tissue samples and different ecological areas. The heatmap representation of the results showed that the root samples, i.e., CDR and QXR clustered together, exhibiting a relatively similar community structure (Fig. 3c). The top 30 genera (i.e., those with relative abundance > 1%) were also selected to make a clustering heatmap (Fig. 3d). At the genus level, *Auricularia* and *Mycena* were the dominant genera in root samples (CDR and QXR), while *Mortierella, Pestalotiopsis*, *Cladophialophora Agaricomycetes*, and *Herpotrichiellaceae* were more abundant in the QXR samples than in CDR samples. The results of fungal communities showed that CDR and QXR samples clustered together, as did CDS and QXS, CDY and QXY, exhibiting a relatively similar community structure. The results hinted that the origin of endophytic fungi in roots is different from that in leaves and stems.

### Alpha diversity analyses of the endophytic fungal communities in H. serrata. from different ecological areas

**Fig. 4:**
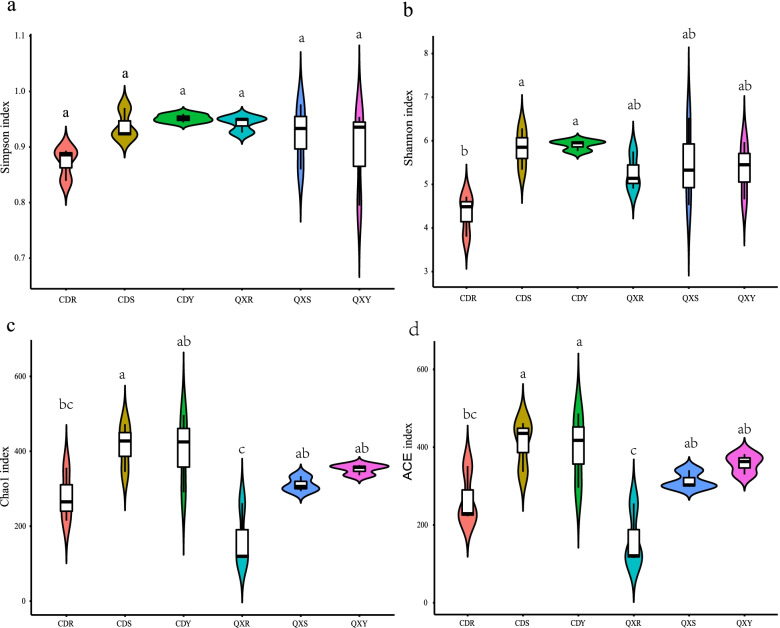
Violin of the alpha diversity indices of the endophytic fungal communities of *H. serrata *Simpson (**a**) Chao1 (**b**) ACE (**c**) and Shannon (**d**) indices. Each violin represents the distribution of diversity present in three replicates (*n* = 3). Different lower-case letters represented a significant difference (*P* < 0.05) was assessed by one-way ANOVA followed by the Duncan's multiple range test.

Alpha diversity analyses, including Shannon, Simpson, Chao1 and ACE indices, were conducted by Wilcoxon rank-sum test, to characterize differences in fungal community abundances and diversities in different groups. The results of the alpha diversity analysis of the fungal communities indicated that the Simpson index of the six samples was not significant (Fig. 4a). Specifically, the Shannon index of CDS and CDY samples was significantly higher than CDR samples (*P* < 0.05) (Fig. 4b). The Chao1 index of the CDS samples was significantly higher than those of CDR and QXR samples, while the Chao1 index of the QXR samples was significantly lower than those of except five samples (*P*< 0.05) (Fig. 4c). The ACE index of CDR samples was significantly lower than those of CDS and CDY samples, and the ACE index of QXR samples was significantly lower than that of QXS and QXY samples (*P* < 0.05) (Fig. 4d).

### Beta diversity analyses of the endophytic fungal communities in *H. serrata*. from different ecological areas

**Fig. 5:**
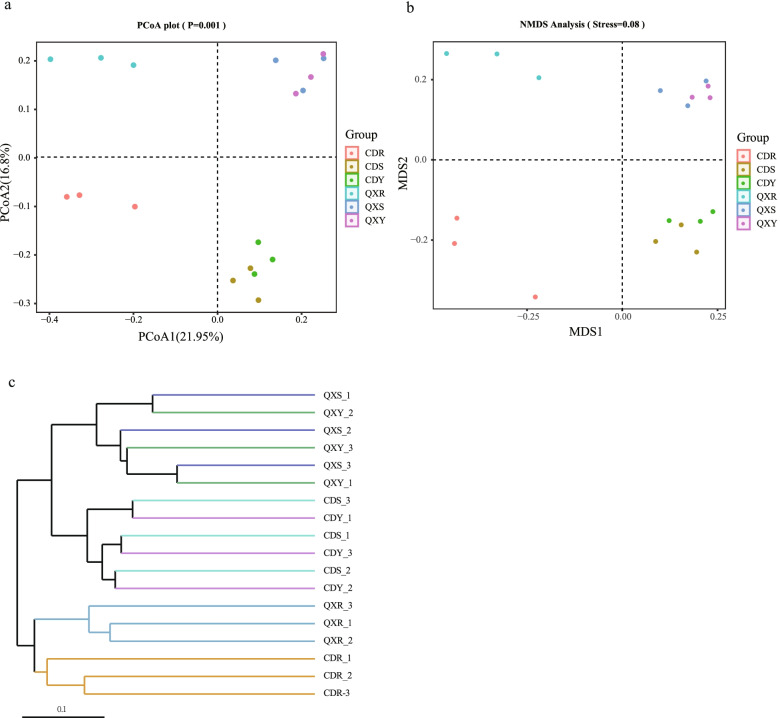
Beta diversity analysis of endophytic fungal community based on unweighted UniFrac distance for the *H. serrata* samples. **a**. Principal coordinate analysis (PCoA) plot. **b**. Non-Metric Multi-Dimensional Scaling (NMDS) analysis. **c**. UPGMA tree of different fungal community structures at the genus level in the different samples

Principal coordinate analysis (PCoA), used to revealed variations among different *H. serrata* samples, was performed based on the unweighted Unifrac distance matrix. In the PCoA result showed that the first axis and the second axis explained 21.95% and 16.8% of the data’s variability, respectively. (Fig. 5). It revealed that the structures of the endophytic fungal communities of the stem and leaf samples (CDS and CDY, QXS and QXY) were relative similar. In contrast, the endophytic fungal communities in roots (CDR and QXR) were distinctly separated from those of the stems and leaves (CDS and CDY, QXS and QXY). Interestingly, the endophytic fungal communities of same tissue samples (CDR and QXR, CDS and QXS, CDY and QXY) from different ecological areas were distinctly separated (Fig. 5a). Similar results were found in Non-metric multidimensional scaling (NMDS) analysis, hinting that plant tissues and ecological areas affected the structure and diversity of endophytic fungal communities fin *H. serrata* (Fig. 5b).

UPGMA tree, conducted by unweighted unifrac method based on the genus level, revealed that two main different clusters were observed. Among them, the endophytic fungal communities from leaf and stem samples (CDS and CDY, QXS and QXY) clustered together, while the root samples (CDR and QXR) clustered alone and distinctly separated from the stem and leaf samples. Otherwise, the endophytic fungal communities of the leaf samples and stem samples in same ecological areas (CDS to CDY, QXS to QXY) were more similar than that of different ecological areas (CDS to QXS, CDY to QXY) (Fig. 5c). The results suggested that the endophytic fungal communities of the root samples might have species-specific, and those of the leaf and stem samples probably have ecological specificity.

**Fig. 6:**
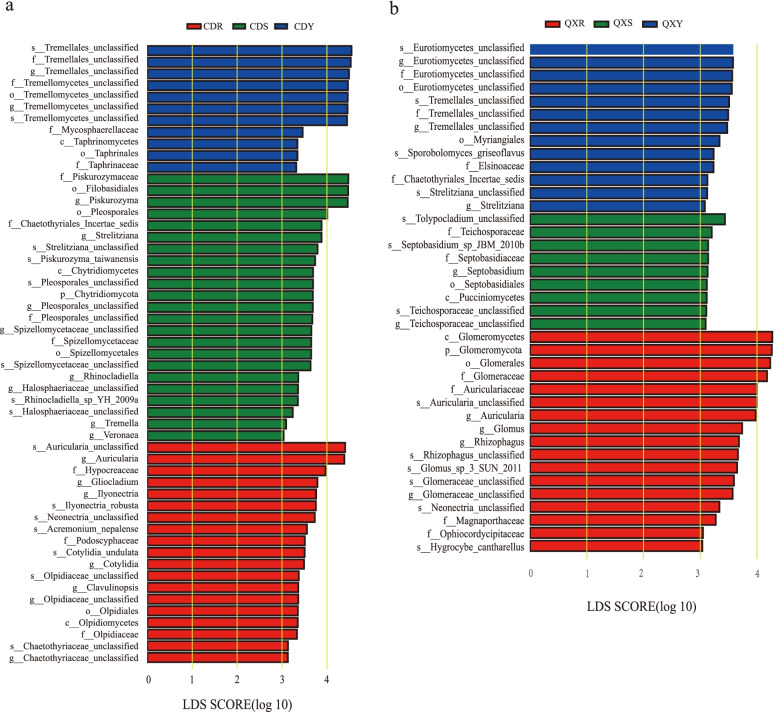
Linear discriminant analysis effect size (Lefse) analysis of differentially abundant taxonomic clades in fungal communities from Chengdu (**a**) and Qixianhu(**b**) with an LDA score higher than 4.0. The figure shows the taxa with an LDA score greater than 4.0. The length of the horizontal bars represents the effect size for each taxon (LDA score), and different colors represent different grouped taxa

Linear discriminant analysis effect size (LEfSe) analysis was used to discover the biomarkers of endophytic fungal community among the six samples from two ecological areas. A total of 53 biomarkers were discovered and employed to discern significant differences among six samples with an LDA score greater than 3.0. The result showed that more taxa with statistically significant abundance in Chengdu samples than in Qixianhu samples at the genus level. Among them, the CDY samples contain more Tremellales_unclassified, and Tremellomycetes_unclassified, CDS samples contain more *Piskurozyma*, *Strelitziana*, Pleosporales_unclassified, Spizellomycetaceae_unclassified, *Rhinocladiella*, Halosphaeriaceae_unclassified, *Tremella,* and *Veronaea*. And *Auricularia*, *Gliocladium*, *Ilyonectria*, *Cotylidia*, *Clavulinopsis*, Olpidiaceae_unclassified, and Chaetothyriaceae_unclassified are more abundant in CDR samples (Fig. 6a). In contrast, *Auricularia*, *Glomus*, *Rhizophagus,* and Glomeraceae_unclassified were significantly enriched in QXR samples, while Eurotiomycetes_unclassified, Tremellales_unclassified, and *Strelitziana* were more abundant in QXY samples. And *Septobasidium* and Teichosporaceae_unclassified were more abundant in QXS samples (Fig. 6b).

### Correlation Analysis between fungal endophytes diversity and Hup A content

**Fig. 7:**
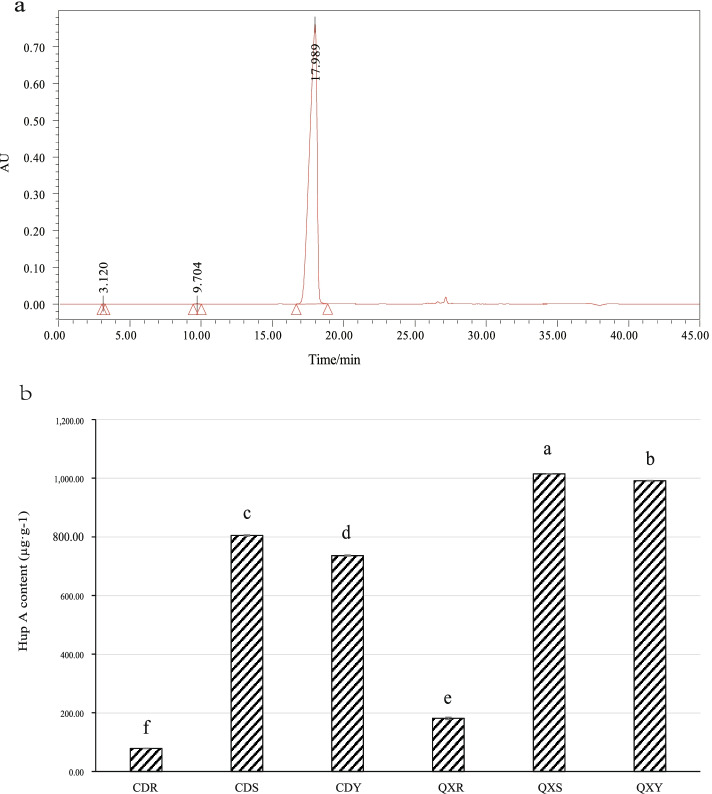
HPLC analysis of the Hup A standard (**a**) and Hup A contents in different samples (**b**). The retention time for Hup A standard was 17.989 min. Different letters on the bars indicate significant differences among all means in different treatments using a one-way ANOVA followed by the Duncan's multiple range test (*P* < 0.05)

**Fig. 8:**
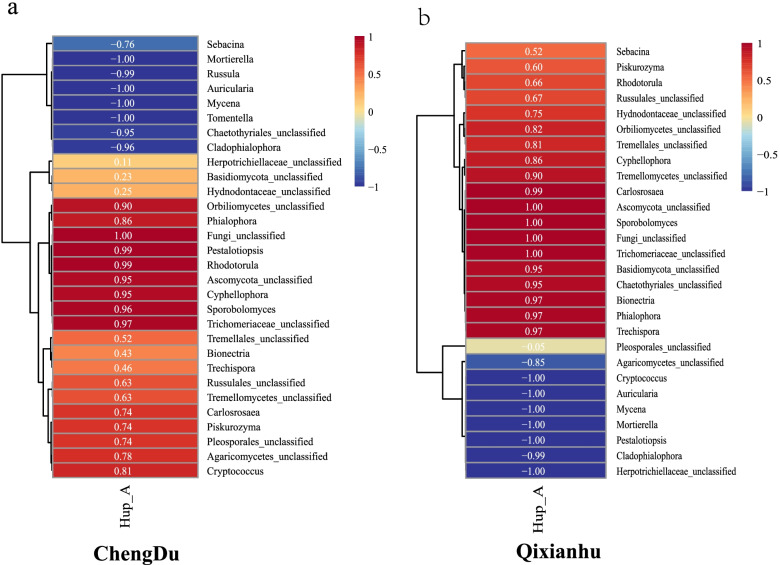
The correlation of Hup A content with endophytic fungi community from Chengdu samples (**a**) and Qixianhu samples (**b**). The correlation index (CI) was shown in figures

The content of Hup A in different samples was quantified by high performance

liquid chromatography (HPLC) (Fig. 7). The retention time of standard Hup A was 17.989 min, and the HPLC spectrum of Hup A standard is shown in Fig. 7a. The results showed that the content of Hup A in roots (CDR and QXR) was significantly lower than that in stems and leaves. The Hup A content in Qixianhu samples was significantly higher than that in Chengdu samples, hinting that Hup A content might have variety specificity (Fig. 7b). The correlation between the top 30 OTUs of endophytic fungal community and Hup A content is depicted using Pearson heat map (Fig. 8). There were 7 genera ( Fungi_unclassified, *Pestalotiopsis*, *Rhodotorula*, Ascomycota_unclassified, *Cyphellophora, Sporobolomyces*, and Trichomeriaceae_unclassified) were significantly and positively correlated to Hup A content of Chengdu samples(CI ≥ 0.95). At the same time, there were 7 genera (Mortierella, Russula, Auricularia, Mycena, Tomentella, Chaetothyriales_unclassified, and *Cladophialophora*) were significantly and negatively correlated to Hup A content of Chengdu samples (CI ≤—0.95) (Fig. 8a). On the other hand, there were 10 genera (*Carlosrosaea,* Ascomycota_unclassified, *Sporobolomyces,* Fungi_unclassified, Trichomeriaceae_unclassified, Basidiomycota_unclassified, Chaetothyriales_unclassified, *Bionectria, Phialophora,* and *Trechispora*) were significantly and positively correlated to Hup A content of Qixianhu samples (CI ≥ 0.95).There were 7 genera (Fungi_unclassified, *Pestalotiopsis, Rhodotorula,* Ascomycota_unclassified, *Cyphellophora, Sporobolomyces,* and Trichomeriaceae_unclassified) were significantly and negatively correlated to Hup A content of Chengdu samples(CI ≤ -0.95) (Fig. 8b). Of which, there are 6 genera (Ascomycota_unclassified, *Cyphellophora,* Fungi_unclassified, *Sporobolomyces,* and Trichomeriaceae_unclassified) were significantly and positively correlated to Hup A content in all two areas, whereas, there are 6 genera (*Auricularia, Cladophialophora, Cryptococcus, Mortierella,* and *Mycena*) were significantly and negatively correlated to Hup A content in all two areas. These genera, which showed positively or negatively correlated Hup A content of in all two areas, may probably have species-specific. However, those genera which showed positively or negatively correlated Hup A content in only one area may probably have eco-environmental specificity.

**Fig. 9:**
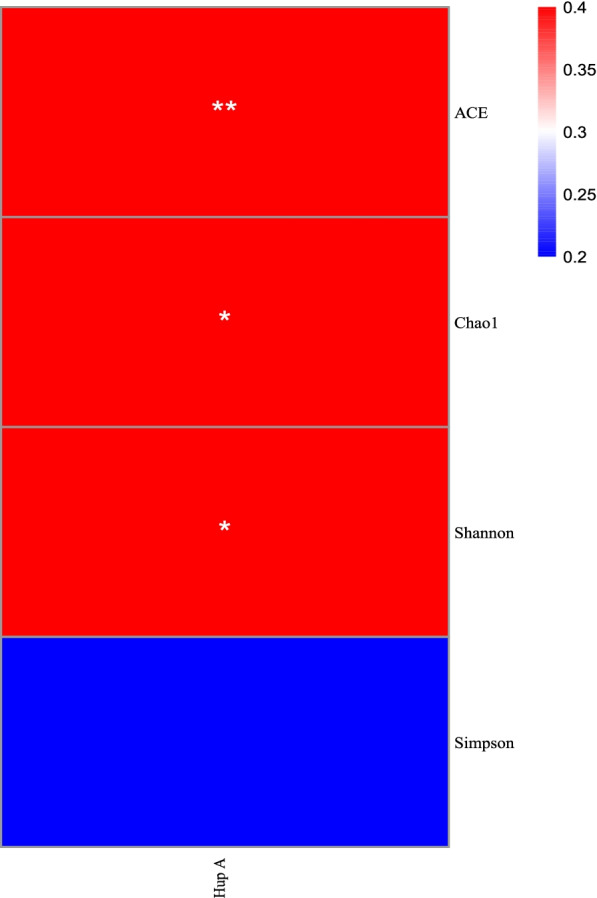
Pearson correlation analysis between diversity of endophytic fungi and Hup A content. Note: ** indicate the differences are significant at *P* < 0.01 and * indicate the differences are significant at *P* < *0.05*

Pearson correlation analysis showed that the Hup A contents were significantly positively correlated with endophytic fungal ACE index, and positively correlated with Chao1 and Shannon’s diversity index of endophytic fungi in *H. serrata* (Fig. 9).

## Discussion

In this study, a total of 2521 endophytic fungal OTUs were detected from six tissue samples of *H. serrata* derived from two ecological areas. Alpha diversity analysis revealed that the richness and diversity of the endophytic fungal communities of different tissues in *H. serrata* were various significantly (*P* < 0.05), with greatest richness and diversity in leaf samples, followed by stem and roots. In previous reports, the endophytic fungi in leaves of the medicinal plant *Mansoa alliacea* were more richness than those of in stems [25]. Similar results have also been reported by other reports [23, 24, 26–28].

The compositions of endophytic fungi in the root, stem, and leaf of *H. serrata* were analyzed at the phylum and genus levels. At the phylum level, the endophytic fungi of *H. serrata* included members of the *Ascomycota*, *Basidiomycota*, *Zygomycota*, *Glomeromycota*, *Chytridiomycota*, *Olpidiomycota*, *Mucoromycota*, *Mortierellomycota*, and a small number of unidentified fungi. Of these groups, the *Ascomycota* and *Basidiomycota* phylum were the most abundant overall, and the *Chytridiomycota was* relatively abundant in leaf and stem samples than in root samples. The results are similar to previous reports that dominant endophytic fungi communities in *H. serrata* and are consistent with other reports of endophytic fungi in medicinal plants [28, 29].

At the genus level, a total of 430 genera were identified, and the compositions and proportions of the genera were significantly different in different tissues from the two ecological areas. The genus *Ascomycota* and *Piskurozyma* were more abundant in leaf and stem samples (CDY, CDS, QXY, and QXS) than in root samples (CDR and QXR). On the contrary, *Cladophialophora* and *Mycena* were more abundant in root samples than in leaf and stem samples. These genera displayed tissue-specific characteristics. The *Sebacina* was the second dominant genus in Chengdu samples (CDR, CDS, and CDY), but it was hardly detected in Qixianhu samples (QXR, QXS, and QXY). The same results were also found in *Russula* and *Mycena* genus. Other studies showed that the *Sebacina* genera could be detected in all detected samples in Hunan Province [23]. It has been reported that the endophytic fungus *Sebacina* genera could increase the survival of regenerated tobacco plantlets and increase the root and shoot biomass of *Zea mays*, tobacco, *Petroselinum crispum, Spilanthes calva*, and *Withania somnifera* [30, 31]. The *Sebacina* genera showed different distribution characteristics, hinting that *Sebacina* may probably have ecological-specific rather than species-specific. On the other hand, *Agaricomycetes*, *Pleosporales*, *Trechispora*, and *Rhodotorula* were more abundant in Qixianhu samples than in Chengdu samples. These genera also showed ecological-specific characteristics. In a previous study, Fan et al. [23] report that *Cladosporium*, *Oidiodendron*, *Phyllosticta*, *Sebacina*, and *Ilyonectria* were dominant genera. And in another report, Cui et al. [24] indicated that *Cladophialophora* is the dominant genera in endophytic fungal strains based on potato dextrose agar (PDA) medium cultured method. The composition and frequency of endophytic fungal taxa varied greatly among different areas, which might be related to altitude, exposure, related vegetation type of tissue, phase of tissue, and mean annual temperature of different production areas, rhizosphere soil, and climate [14, 32, 33].

Furthermore, traditional microbial research methods, such as isolation and culture, are restricted by various factors, which usually leads to underestimation of microbial community composition and diversity [34, 35]. The application of high-throughput sequencing technology in plant rhizosphere and in vivo microbial research effectively avoids the disadvantages of traditional methods and it is an effective method for acceptable microbial research [21, 36].

The results of the alpha diversity analysis of the fungal communities indicated that there were no significant differences in the Simpson among the six groups. Furthermore, there were also no significant differences in the Simpson, Chao1, ACE, and Shannon indices among the same organs from different ecological areas, i.e., root (CDR and QXR), stem (CDS and QXS), and leaf (CDY and QXY). While, Shannon indices showed that the diversity of endophytic fungi in the Chengdu root sample (CDR) was lower than that of the rest five groups (CDS, CDY, QXR, QXS, and QXY). The Chao1, and ACE indices showed that the diversity of endophytic fungi in root samples was the lowest among all samples. In total, diversity and abundance of endophytic fungal community in the roots of *H. serrata* was lower than that of in the leaves and stems.

Beta diversity analyses are usually used to compare microbial community composition among environmental gradients [37, 38]. In our study, the stem and leaf samples from the same areas (CDS and CDY, QXS and QXY) were grouped together based on similarity in endophytic fungal community composition and diversity (Fig. 4a, b). However, the root samples from the two areas were more similar than they were to the stem and leaf samples (Fig. 4a, b). The communities of endophytic fungi within the stems were similar to the communities within leaves at all taxonomic levels. Which is also a reasonable agreement with other studies of different plants that contain a higher species richness and diversity in the leaves and stems than in the roots [39]. Since the *H. serrata* is a perennial plant with a relatively larger surface areas in leaves and stems than in roots. This leads to more opportunities for leaves and stems to contact with air and microbe during growth and development. At the same time, leaf and stem are rich in nutrients and also provides rich nutritional conditions for the reproduction of many endophytic fungi. Which is probably one of the main reasons of more abundance and higher diversity of fungal communities in leaves and stems than those in roots.

Several studies reported that the Hup A compound is produced by many endophytic fungi, including *Aspergillus*, *Colletotrichum*, and *Fusarium* strains, which offers an alternative method to reduce the burden on wild *H. serrata* plant and meet the increasing demand for the compound [17]. Our report revealed that the content of Hup A was different in all detected organs of *H. serrata*. In summary, the highest content was found in the leaves and stems, while the roots had slightly smaller quantities, whereas the Hup A contents are various among the different areas. The results are consistent with those of previous results [4, 7]. Furthermore, the Hup A content in the Qixianhu samples was slightly higher than that in the Chengdu samples. Pearson correlation analysis showed that 6 genera were positively correlated with Hup A content in the two areas, and 6 genera were negatively correlated with Hup A content in the two areas, indicating that these genera have species-specificity. In contrast, others genera may have ecological environment specificity*.* Pearson correlation analysis also showed that the Hup A contents were significantly positively correlated with ACE index, and positively correlated with Chao1 and Shannon’s index of endophytic fungi in *H. serrata* (Fig. 9). It is known that Hup A is an alkaloid extracted from herbal plant *H. serrata.* Numerous studies have shown that alkaloids are a class of secondary metabolites that respond to biotic and abiotic adversity stress. Many alkaloids have a bitter taste, so they have obvious toxicity or antifeedant properties to avoid animal damage. Another part has antibacterial function, which can help resist the invasion of microorganisms. Some alkaloids are also involved in habitat stress tolerance. As a perennial plant, *H. serrata* usually grows in the shade of high altitudes and needs to resist pests, pathogens, and harsh environments. Stems and leaves that have been exposed to air for a long time may need to produce more Hup A to help resist endophytic fungi and environmental stress. This may be the reason why the content of Hup A in leaves and stems is much higher than that in roots.

Up to now, totally about 13 fungal genera have been reported to produce Hup A [40, 41]. Of which 8 genera were found in our report and accounted for 61.5%. Remain 5 of the reported Hup A-producing endophytic fungal genera could not be detected in this report, which is probably because the planting environment and developmental stage of the host plant affect the endophytic fungi community. Thus, our study sets the ground for new insights into the diversity and composition of endophytic fungi in *H. serrata,* as well as the interaction between the endophytic fungi community and Hup A production*.*

## Materials and methods

### Experimental materials and methods

The natural populations of *H. serrata* materials were collected from two ecological areas, Chengdu city, Sichuan Province, China (30◦34’N, 103◦64’E) (voucher No. HS202104201) and Qixianhu area, Pan’an county, Zhejiang Province, China (28◦88’N, 120◦55’E) (voucher No. HS202104201), respectively. These two ecological areas almost have similar latitudes and climates, but the distance is more than 2,000 km. Two samples were identified as *H. serrata* by Pan Lanlan, senior engineer of Dapanshan National Nature Reserve. Voucher specimens of these samples were deposited in the Dapanshan National Nature Reserve, Pan’an Zhejiang, China.

To maximize the chance of obtaining endophytic fungi of *H. serrata*, more than 3-years old wild *H. serrata* were collected, which can produce mature spores. Three plants were randomly selected and merged into one sample, and three biological replicates were prepared for each sample. After removing all sporangia, the samples were marked as follows: Chengdu root (marked as CDR_1, CDR_2, CDR_3), stem (marked as CDS_1, CDS_2, CDS_3) and leaf (marked as CDY_1, CDY_2, CDY_3); Qixianhu Wetland Park root (marked as QXR_1, QXR_2, QXR_3), stem (marked as QXS_1, QXS_2, QXS_3) and leaf (marked as QXY_1, QXY_2, QXY_3), respectively.

The whole plant was dug up with roots and soil, kept moist, put in a sterile self-sealing bag, and placed in a refrigerator at 4 ℃. And the material was treated within 24 h. The whole plant of fresh *H. serrata* is continuously washed with tap water until no impurities such as sediment can be seen. After rinsing, the tissue surface of the materials was dried with the aseptic filter paper.

All the tissues of different plants and different sections were surface-sterilized using the following washing steps: 70% (v/v) ethanol for 1 min, 2% (v/v) sodium hypochlorite solution for 5 min, 2.5% (w/v) sodium thiosulfate for 5 min, and rinsing the samples five times with sterile water. And then quickly transported to the liquid nitrogen, and stored at − 80 ℃ for DNA extraction.

### DNA Extraction, Amplicon, and High-Throughput Sequencing

All samples were well-grounded in liquid nitrogen, and 100 mg of each sample was transported to a 2 ml centrifuge tube. Total DNA was extracted using the E.Z.N.A. soil DNA Kit (Omega Bio-tek, Norcross, GA, USA) according the manufacturer’s instructions. The DNA was checked on 1% agarose gel, and DNA concentration and purity were determined with DeNovix DS-11 spectrophotometer (DeNovix Scientific, USA).

The conserved intergenic transcribed spacer 2 (ITS2) region of the fungal rRNA genes was amplified with the primers fITS7 (5'-GTGARTCATCGAATCTTTG-3') and ITS4 (5'-TCCTCCGCTTATTGATATGC-3’) [42]. The PCR mixture (25 µL) contained 1 µL of DNA template (50 ng µL·L^−1^), 12.5 µL Phusion Hot start flex 2X Master Mix(Biolabs, New England), 2.5 µl forward and reverse primers(1 µM for each). The PCR reaction program was as follows: 98 °C for 30 s, followed by 32 cycles at 98 °C for 10 s, 54 °C for 30 s, and 72 °C for 45 s, and a final extension at 72 °C for 5 min.

The resulting products were verified using electrophoresis on 2.0% agarose gels and purified using AMPure XT beads (Beckman Coulter Genomics, Danvers, MA, USA) and quantified with a Qubit instrument (Invitrogen, USA). The amplicon pools were prepared for sequencing, and the size and quantity of the amplicon library were assessed using an Agilent 2100 Bioanalyzer (Agilent, USA) and the Library Quantification Kit for Illumina (Kapa Biosciences, Woburn, MA, USA), respectively. The libraries were sequenced on the NovaSeq P6000 platform at LC-Bio Technology Co., Ltd, Hang Zhou (Hangzhou, China), according to the manufacturer’s recommendations.

### Sequence processing and data analysis

Cutadapt software (v1.9) was used to assign and truncate paired-end reads based on their unique barcodes and primer sequences. Paired-end reads were merged using the PEAR R software(v0.9.6). Fqtrim software (v0.94) was used to perform quality filtering of the raw tags to obtain clean tags. Chimeric sequences were filtered using the Vsearch software (v2.3.4). After dereplication, the feature table and the feature sequence were obtained using DADA2, which were further clustered into operational taxonomic units (OTUs) with 97% pairwise identity. The function of fungi was predicted and analyzed by FUNGuild [43].

Alpha diversity and beta diversity were calculated by QIIME2 software, in which the same number of sequences were extracted randomly by reducing the number of sequences to the minimum of some samples, and the relative abundance (X fungi count/total count) is used in fungi taxonomy. Alpha diversity and beta diversity were analyzed by the QIIME2 process, and pictures were drawn by R (v3.5.2). The sequence alignment of species annotation was performed by the QIIME2 plugin feature-classifier, and the alignment database was RDP and unite [44]. Linear discriminant analysis impact size (LefSe) was performed to detect differentially abundant taxa across plant organs by using the default parameters.

### Quantification of Hup A in *H. serrata* by HPLC

The Hup A extracts were prepared by the method as reported previously [45]. In brief, The *H. serrata* plant samples were dried at 60 ℃ for 24 h, pulverized, and precisely weighted in 2 g. The powder was dissolved in 40 mL pH = 3.0 HCl and stirred for 10 h at room temperature, then sonicated at 50 ℃ for 1 h. To the filtered solution, 20 mL HCl (pH = 3.0) was added and extracted. After centrifuging again, all the extracts were collected and adjusted the pH value to 9.0 with NH_3_·H_2_O to pH 9.0. Then, the same volume of chloroform was added to the extracts and extracted twice. After that, the chloroform layers were dried by the rotary evaporator and evaporated into dryness at low pressure. Then, obtained residues were dissolved in 10 mL of methanol after evaporation and purified through a filter syringe (0.45 µm) to perform HPLC.

Each sample was extracted and quantified with three replications.

Hup A content was measured by HPLC using a Waters Alliance e2695 (Waters, WATERS Corporation, MA, USA) with a Waters SunFire C18 column (4.6 mm × 250 mm, 5um; WATERS Corporation, MA, USA). The temperature of the column compartment was maintained at 25 °C. The flow rate was 1.0 mL/min using 80 mM ammonium acetate (pH 6.0)–methanol (65:35, v/v) as the mobile phase. Samples of 20 μL were loaded for detection at 310 nm [45]. The Hup A contents in the samples were quantified by using the standard curve generated from the Hup A standard (purchased from Chengdu Desite Biotechnology Co. Ltd., Chengdu, China), which is linear in the range of 5.2–52 µg/mL and with r^2^ = 0.9999.

## Data Availability

All raw sequencing data generated and analyzed in this article are available at the National Center for Biotechnology Information(NCBI). Sequence Read Archive under the BioProject PRJNA798846. https://www.ncbi.nlm.nih.gov/Traces/study/?acc=PRJNA798846, SRA accession: SRR17679188- SRR17679205.
